# Glucagon-like Peptide-1 Receptor Agonists and Dual Incretin Therapies Across HFpEF and Cardiovascular Outcomes Trials in Patients with Obesity

**DOI:** 10.3390/medicina62081446

**Published:** 2026-07-25

**Authors:** Andrej Belančić, Sanja Klobučar, Kristina Skroče, Andreea Ciudin, Josip Anđelo Borovac

**Affiliations:** 1 Department of Basic and Clinical Pharmacology and Toxicology, Faculty of Medicine, University of Rijeka, 51000 Rijeka, Croatia; 2 Department of Endocrinology, Diabetes and Metabolic Diseases, Clinical Hospital Centre Rijeka, 51000 Rijeka, Croatia; sanja.klobucarm@gmail.com; 3 Department of Internal Medicine, Faculty of Medicine, University of Rijeka, 51000 Rijeka, Croatia; 4 Faculty of Medicine, University of Rijeka, 51000 Rijeka, Croatia; kristina.skroce@gmail.com; 5 Diabetes and Metabolism Research Group, VHIR, Endocrinology Department, Vall d’Hebron University Hospital, Autonomus University of Barcelona, 08035 Barcelona, Spain; andrea.ciudin@vallhebron.cat; 6 Department of Cardiovascular Diseases, University Hospital of Split, 21000 Split, Croatia; josip.borovac@me.com; 7 Department of Pathophysiology, School of Medicine, University of Split, 21000 Split, Croatia

**Keywords:** cardiovascular outcomes, GLP-1 receptor agonists, heart failure, obesity, semaglutide, tirzepatide

## Abstract

Glucagon-like peptide-1 receptor agonists (GLP-1 RAs) and emerging dual incretin therapies have demonstrated substantial effects on weight reduction and metabolic improvement, with growing evidence supporting favorable cardiovascular outcomes. This narrative review provides a focused overview of key cardiovascular outcomes trials (CVOTs) and HFpEF-oriented clinical studies evaluating GLP-1 RA and dual incretin therapies in patients living with obesity, including completed and ongoing investigations. We summarize trial characteristics, critically appraise their outcomes, and briefly discuss mechanistic pathways potentially linking incretin-based therapies to cardiovascular and HFpEF-related benefits. Because available data derive from heterogeneous study designs, we explicitly distinguish evidence from type 2 diabetes cardiovascular outcomes trials, obesity-without-diabetes cardiovascular outcomes trials, HFpEF-specific randomized trials, mechanistic substudies, ongoing trials, and real-world observational analyses. We also situate incretin-based therapies within contemporary obesity, diabetes, cardiovascular, and HFpEF guideline-directed care. Finally, we identify current knowledge gaps, future research priorities, and innovative approaches needed to refine personalized therapeutic strategies and optimize patient-centered outcomes in obesity-associated HFpEF.

## 1. Introduction

Obesity is a chronic, relapsing disease defined by the World Health Organization as abnormal or excessive fat accumulation that presents a risk to health, commonly operationalized in adults as a body mass index (BMI) ≥ 30 kg/m^2^ [1]. Over recent decades, its prevalence has increased dramatically, reaching global pandemic proportions with no clear evidence of stabilization [2,3]. In 2022, more than 1 billion individuals were living with obesity worldwide, with prevalence increasing in nearly every country [4]. This trajectory is also evident in younger populations: according to the World Obesity Federation’s World Obesity Atlas 2026 [5], an estimated 419 million children and adolescents aged 5–19 years were living with overweight or obesity in 2025, a number projected to increase to 507 million by 2040, when more than one in four children and adolescents will be affected by high BMI.

Beyond its clinical consequences, obesity imposes a substantial and escalating economic burden through increased healthcare expenditure, reduced productivity, and long-term disability [6]. If current trends persist, the global economic impact of overweight and obesity is projected to reach approximately 3.29% of global gross domestic product by 2060, with the greatest increases expected in lower-resource settings [7].

Although BMI remains the most widely used diagnostic metric, it provides limited insight into adiposity distribution, adipose tissue dysfunction, and obesity-related health risk. Accordingly, obesity is increasingly conceptualized as a chronic, systemic disease characterized by multi-organ dysfunction driven by excess and dysfunctional adiposity [2,8]. It is associated with a markedly increased risk of cardiometabolic disease, including type 2 diabetes mellitus (T2DM), hypertension, and cardiovascular disease, as well as musculoskeletal disorders, several malignancies, and autoimmune conditions [9,10,11,12]. In addition, obesity is linked to substantial psychosocial burden, including depression, anxiety, impaired quality of life, and the adverse effects of weight stigma and discrimination [13]. Collectively, these factors contribute to increased all-cause mortality and reduced life expectancy across populations [14,15].

At a mechanistic level, obesity is characterized by pathological expansion of adipose tissue through adipocyte hypertrophy and hyperplasia [16,17]. Adipose tissue is metabolically active and secretes adipokines, cytokines, and chemokines that regulate systemic metabolism and immune responses. Chronic low-grade inflammation is a central pathophysiological feature of obesity, promoting insulin resistance, endothelial dysfunction, and vascular injury, thereby linking excess adiposity to cardiovascular disease [18].

Within this framework, obesity is strongly associated with heart failure with preserved ejection fraction (HFpEF), a phenotype increasingly recognized as an adiposity-driven cardiometabolic syndrome. Obesity-related HFpEF is characterized by plasma volume expansion, low natriuretic peptide concentrations, metabolic dysregulation and systemic inflammation, all of which complicate diagnosis and management. Excess adiposity also contributes to adverse cardiac remodeling and reduces the diagnostic reliability of conventional biomarkers [19]. Beyond HFpEF, obesity is associated with a higher incidence of atherosclerotic and heart failure events, including major adverse cardiovascular events (MACE; typically, cardiovascular death, nonfatal myocardial infarction, or nonfatal stroke) and heart failure hospitalization. These observations highlight a synergistic risk profile in which obesity accelerates HFpEF progression and amplifies the broader burden of cardiovascular events [20].

Glucagon-like peptide-1 receptor agonists (GLP-1 RAs) and dual GLP-1/GIP incretin therapies were initially developed for T2DM but have emerged as effective therapies for obesity management [21]. Growing evidence also suggests potential benefits in obesity-related HFpEF. However, interpretation remains limited by the modest representation of HFpEF populations in individual trials and by the fact that much of the existing cardiovascular evidence derives from cohorts not specifically enriched for obesity. This constrains generalizability, particularly in cardiovascular outcomes trials (CVOTs), where obesity-related pathophysiology may modify therapeutic response. Moreover, extrapolation from non-obese populations is inadequate, and observational data remain susceptible to residual confounding and selection bias.

Current clinical guidelines also provide limited phenotype-specific guidance for the use of GLP-1 RAs and dual incretin therapies in obesity-related HFpEF. Although obesity management is increasingly recognized as clinically relevant in heart failure, guideline recommendations remain constrained by the rapidly evolving evidence base, limited dedicated long-term HFpEF outcome data, and heterogeneity between diabetes CVOTs, obesity CVOTs, and HFpEF-focused trials. As a result, current guidance does not yet fully define which patients with obesity-related HFpEF should be prioritized for incretin-based therapy, how these agents should be integrated with established HFpEF treatments, or whether treatment selection should be based on BMI alone or more specific cardiometabolic and adiposity-related phenotypes.

Therefore, this review provides a focused overview of randomized controlled trials evaluating GLP-1 RAs and dual incretin therapies in populations with obesity, with particular emphasis on HFpEF and cardiovascular outcomes. By synthesizing evidence from CVOTs and HFpEF-specific trials, supplemented by key observational data, we aim to identify critical evidence gaps and priorities for future research.

## 2. Current Guideline Context and Hierarchy of Evidence

Current guidance increasingly recognizes obesity as a chronic, relapsing disease requiring multimodal long-term management. The 2025 WHO guideline conditionally supports GLP-1-based therapies for adults with obesity as part of comprehensive care that also includes nutrition, physical activity, behavioral support, access considerations, and long-term follow-up [22]. The EASO 2026 pharmacological framework similarly places semaglutide and tirzepatide among high-efficacy anti-obesity therapies while emphasizing phenotype, complication burden, tolerability, treatment persistence, and affordability [23].

In patients with T2DM and cardiovascular disease, contemporary guidelines frame GLP-1 receptor agonists and SGLT2 inhibitors as cardiometabolic therapies rather than glucose-lowering agents alone. The ADA Standards of Care 2025 recommend therapies with proven cardiovascular and kidney benefit in patients with T2DM and established or high risk of atherosclerotic cardiovascular disease, heart failure, or chronic kidney disease [24], whereas the ESC diabetes guidelines emphasize GLP-1 receptor agonists with proven cardiovascular benefit in atherosclerotic risk and SGLT2 inhibitors, particularly in heart failure and kidney disease contexts [25]. Chronic coronary syndrome guidance likewise places metabolic and obesity management within global cardiovascular risk reduction [26].

In HFpEF, SGLT2 inhibitors remain the only broadly guideline-directed pharmacological therapy supported across HFpEF and HFmrEF populations [27]. Diuretics, blood-pressure control, management of atrial fibrillation, ischemia, chronic kidney disease and diabetes, exercise rehabilitation, and structured weight management remain foundational. GLP-1 receptor agonists and dual incretin therapies should therefore be viewed as phenotype-directed anti-obesity or cardiometabolic add-on strategies, not as replacements for established HFpEF care.

To avoid conflating different levels of evidence, this review separates five evidence strata: (i) diabetes cardiovascular outcomes trials, which inform agent-level cardiovascular safety and efficacy in T2DM; (ii) obesity-without-diabetes cardiovascular outcomes trials such as SELECT, which more directly address pharmacological obesity treatment and cardiovascular prevention; (iii) HFpEF-specific randomized trials such as STEP-HFpEF and SUMMIT; (iv) mechanistic imaging and biomarker substudies; and (v) observational or real-world analyses, which are clinically informative but hypothesis-generating because of residual confounding and treatment-selection bias.

## 3. Methods

This article was conducted as a narrative review to summarize and critically appraise the available evidence regarding GLP-1 RAs and dual incretin therapies in patients living with obesity, with particular emphasis on CVOTs and HFpEF. A targeted literature search was performed using PubMed and Scopus to identify relevant publications published between 1 January 2021 and 1 May 2026, corresponding to the period encompassing the pivotal trials for semaglutide and tirzepatide for obesity management. Representative search terms included combinations of “GLP-1”, “GIP”, “incretin therapy”, “semaglutide”, “tirzepatide”, “obesity”, “cardiovascular outcomes”, “major adverse cardiovascular events”, “heart failure”, “MACE”, “cardiovascular outcomes trial”, and “HFpEF”. The primary objective of the search was to identify all randomized controlled trials evaluating semaglutide and tirzepatide in patients living with obesity, with a particular focus on CVOTs and HFpEF trials. Relevant observational studies (e.g., STEER trial) were identified through manual searches of reference lists and were included when considered clinically informative, reflecting the authors’ continuous surveillance of emerging evidence in obesity medicine and cardiovascular pharmacotherapy. Earlier landmark publications, including selected CVOTs conducted in patients with T2DM, together with relevant mechanistic studies and guideline documents, were identified through targeted hand-searching to provide historical and scientific context and to highlight the limitations of extrapolating evidence from diabetes populations to patients living with obesity without diabetes. The relevance of identified publications was evaluated independently by the authors according to the objectives of this review. Differences in study inclusion were resolved through discussion until consensus was reached. Evidence was synthesized narratively, with primary emphasis on randomized controlled trials, while observational studies, mechanistic investigations, and guideline documents were incorporated occasionally to support interpretation of clinical findings and identify current evidence gaps. As a narrative review, this work did not follow the Preferred Reporting Items for Systematic Reviews and Meta-Analyses (PRISMA) framework, and no formal risk-of-bias assessment or quantitative meta-analysis was performed.

## 4. Cardiovascular Outcome Trials of GLP-1 RAs and Dual Incretin Therapies in Obesity

Long-acting GLP-1 RAs have demonstrated cardiovascular (CV) safety and, in several trials, cardiovascular benefit in patients with T2DM. In this section, diabetes CVOTs are used to define agent-level cardiovascular safety and efficacy in T2DM, whereas obesity-without-diabetes CVOTs are treated as the more directly relevant evidence base for cardiometabolic risk reduction in people living with obesity without diabetes. Landmark CVOTs, including LEADER (liraglutide), SUSTAIN-6 (semaglutide) and REWIND (dulaglutide) reported reductions in major adverse cardiovascular events (MACE), supporting the role of incretin-based therapies as key cardiometabolic agents [28,29,30]. More recently, the head-to-head, active-comparator SURPASS-CVOT demonstrated the non-inferiority of tirzepatide compared with dulaglutide with respect to MACE, further supporting the cardiovascular safety of tirzepatide in patients with T2DM [31]. However, extrapolation of these findings to individuals living with obesity without diabetes is not appropriate, given substantial differences in baseline metabolic profiles, cardiovascular risk, and underlying disease biology. Therefore, interpretation of CV benefit must remain contextualized within the studied populations, with careful consideration of external validity and generalizability [32]. To date, the only completed CVOT specifically conducted in individuals with overweight or obesity without diabetes is SELECT [33].

The SELECT trial represents the only completed cardiovascular outcomes trial specifically conducted in individuals living with overweight or obesity without diabetes. In this multicenter, randomized, double-blind, placebo-controlled, event-driven superiority trial, adults aged 45 years or older with a BMI of at least 27 kg/m^2^ and established cardiovascular disease were enrolled [33]. Established cardiovascular disease was defined as a history of myocardial infarction, ischemic stroke, or symptomatic peripheral arterial disease. Key exclusion criteria included any history of diabetes, glycated hemoglobin (HbA1c) ≥ 6.5%, recent use of glucose-lowering therapies or GLP-1 RAs, New York Heart Association (NYHA) functional class IV heart failure, end-stage kidney disease or dialysis, and recent cardiovascular or planned revascularization procedures.

A total of 17,604 participants were randomized in a 1:1 ratio to receive once-weekly subcutaneous semaglutide 2.4 mg or placebo. Semaglutide was initiated at 0.25 mg weekly and escalated every 4 weeks to 0.5 mg, 1.0 mg, 1.7 mg, and finally 2.4 mg over a 16-week titration period. The mean duration of exposure was 34.2 ± 13.7 months, and the mean follow-up was 39.8 ± 9.4 months.

The primary endpoint, a composite of cardiovascular death, nonfatal myocardial infarction, or nonfatal stroke, occurred in 6.5% of participants in the semaglutide group compared with 8.0% in the placebo group, corresponding to a hazard ratio (HR) of 0.80 (95% confidence interval [CI], 0.72–0.90; *p* < 0.001). Cardiovascular death occurred in 2.5% versus 3.0% of participants in the semaglutide and placebo groups, respectively (HR, 0.85; 95% CI, 0.71–1.01; *p* = 0.07), and did not meet the prespecified hierarchical threshold for statistical significance. Consequently, formal superiority testing of subsequent secondary endpoints was not performed. Nevertheless, exploratory analyses demonstrated consistent reductions in heart failure events (HR, 0.82; 95% CI, 0.71–0.96) and all-cause mortality (HR, 0.81; 95% CI, 0.71–0.93).

Overall, SELECT provided robust evidence that pharmacological weight reduction treatment with semaglutide 2.4 mg reduces cardiovascular events in individuals with overweight or obesity and established cardiovascular disease, but without diabetes. Importantly, the trial demonstrated that cardiovascular risk reduction in this population extends beyond glycemic improvement and is likely mediated through multiple mechanisms, including weight loss, blood pressure reduction, anti-inflammatory effects, and broader metabolic modulation. However, the findings are primarily applicable to secondary prevention populations, and the relative contributions of weight loss versus direct drug-specific effects remain incompletely defined [33].

In contrast, SURMOUNT-MMO (NCT05556512) is an ongoing phase 3, randomized, double-blind, placebo-controlled, event-driven trial designed to evaluate the effect of tirzepatide on morbidity and mortality in adults living with obesity without diabetes [34]. The trial plans to enroll approximately 15,000 participants across multiple international sites. Eligible individuals are aged 40 years or older, have a BMI of at least 27 kg/m^2^, and have either established cardiovascular disease or multiple cardiovascular risk factors, thereby including both secondary and primary prevention populations in an approximate 2:3 ratio.

Participants are randomized to receive once-weekly subcutaneous tirzepatide or placebo. Tirzepatide is initiated at 2.5 mg and titrated every 4 weeks over a 24-week period to a maximum dose of 15 mg or the highest tolerated dose. The study includes a screening period, a dose-escalation phase, and a long-term maintenance period, with treatment duration determined by event accrual. The primary endpoint is a five-component composite of all-cause mortality, nonfatal myocardial infarction, nonfatal stroke, coronary revascularization, and heart failure events requiring hospitalization or urgent care. Key secondary endpoints include three-point MACE (MACE-3), incident T2DM, renal function decline assessed by estimated glomerular filtration rate (eGFR) slope, and functional outcomes assessed using the 36-item Short Form Health Survey (SF-36) physical functioning domain. The trial also includes a broad range of additional cardiometabolic, renal, hepatic, and patient-reported outcomes. The SURMOUNT-MMO is particularly important because it is the first incretin-based cardiovascular outcomes trial designed to systematically evaluate both primary and secondary cardiovascular prevention in individuals living with obesity without diabetes. Its broader endpoint structure, including heart failure and revascularization events, may provide a more comprehensive assessment of cardiovascular benefit beyond classical MACE definitions. However, interpretation of future results will require careful consideration of endpoint heterogeneity and the relative clinical weighting of different event types [34].

Finally, emerging real-world evidence, including the STEER study, has also evaluated potential differences between semaglutide and tirzepatide with respect to cardiovascular outcomes in individuals with overweight or obesity and established atherosclerotic cardiovascular disease but without diabetes [35]. In this large retrospective analysis of a United States (US) claims database, semaglutide was associated with statistically significant reductions in recurrent cardiovascular events compared with tirzepatide, including a 29% lower risk of recurrent MACE-3 (HR, 0.71; *p* = 0.046) and a 22% lower risk of recurrent five-point MACE (MACE-5) (HR, 0.78; *p* = 0.040). In the per-protocol analysis, semaglutide remained associated with significantly lower risks of both MACE-3 (HR, 0.43; *p* = 0.005) and MACE-5 (HR, 0.57; *p* = 0.003) compared with tirzepatide [35]. Although these findings provide valuable real-world insight into potential differences between incretin-based therapies, they should be interpreted cautiously because retrospective observational analyses are inherently susceptible to residual confounding, selection bias, and treatment-selection effects. Therefore, these data should be considered hypothesis-generating and cannot replace evidence from randomized controlled trials.

Recent evidence also broadens the cardiovascular discussion beyond obesity and HFpEF. In a type 1 diabetes population, Xu et al. reported associations between GLP-1 receptor agonist use and major cardiovascular and kidney outcomes [36], and Wu et al. described a real-world propensity analysis evaluating GLP-1 receptor agonists in relation to cancer therapy-related cardiac dysfunction [37]. These studies are clinically relevant but outside the core obesity-CVOT/HFpEF evidence stream; therefore, they should be regarded as contextual and hypothesis-generating rather than as evidence to guide treatment selection.

Based on currently available evidence, semaglutide has the strongest cardiovascular outcomes evidence among incretin-based therapies for patients living with obesity and elevated cardiovascular risk, primarily because of the robust randomized data from the SELECT trial [33], whereas SURMOUNT-MMO remains ongoing [34]. This interpretation is also consistent with the updated European Association for the Study of Obesity (EASO) framework for the pharmacological treatment of obesity and its complications [23]. The results of SURMOUNT-MMO are highly anticipated, as they will provide dedicated randomized cardiovascular outcomes data for tirzepatide in patients living with obesity. However, the most clinically informative approach for future decision-making would be a direct head-to-head cardiovascular outcomes trial comparing semaglutide and tirzepatide in this population.

## 5. GLP-1 RAs and Dual Incretin Therapies Across HFpEF Trials in Patients with Obesity

HFpEF is increasingly recognized as a cardiometabolic syndrome strongly linked to obesity, systemic inflammation, congestion, and impaired exercise capacity. In this context, GLP-1 RAs and dual incretin therapies have reshaped therapeutic expectations by targeting excess adiposity and several obesity-related pathophysiological pathways. Among randomized trials, STEP-HFpEF and SUMMIT provide the strongest evidence to date for incretin-based therapy in patients with obesity-related HFpEF.

The STEP-HFpEF trial evaluated once-weekly subcutaneous semaglutide 2.4 mg in patients with obesity-related HFpEF. The study population included adults with HFpEF, defined by left ventricular ejection fraction (LVEF) ≥ 45%, obesity, defined as BMI ≥ 30 kg/m^2^, NYHA functional class II–IV symptoms, and impaired quality of life. Participants were randomized to receive semaglutide 2.4 mg or placebo in a randomized, double-blind, placebo-controlled design over 52 weeks. The dual primary endpoints were change in the Kansas City Cardiomyopathy Questionnaire Clinical Summary Score (KCCQ-CSS) and percentage change in body weight at week 52. Confirmatory secondary endpoints included changes in 6-minute walk distance (6MWD), C-reactive protein (CRP) and hierarchical composite heart failure outcomes [38].

The LVEF criterion in STEP-HFpEF (≥45%) means that a subset of enrolled patients may fall within the HFmrEF range (41–49%) rather than strictly within conventional HFpEF (≥50%). Accordingly, STEP-HFpEF results are best interpreted as evidence across obesity-related symptomatic heart failure with preserved or mildly reduced systolic function, whereas SUMMIT, which required LVEF ≥ 50%, more specifically targeted HFpEF. This distinction is important because HFmrEF may differ from HFpEF in ischemic burden, reverse-remodeling potential, neurohormonal activation, prognosis, and treatment responsiveness [39].

In the intention-to-treat (ITT) population of 529 randomized participants, semaglutide improved KCCQ-CSS by 16.6 points compared with 8.7 points with placebo, corresponding to an estimated between-group difference of +7.8 points. Body weight decreased by 13.3% with semaglutide compared with 2.6% with placebo, corresponding to an estimated between-group difference of −10.7%. The 6MWD improved by 21.5 m with semaglutide compared with 1.2 m with placebo, respectively. The mean percentage change in CRP was −43.5% with semaglutide and −7.3% with placebo, corresponding to an estimated treatment ratio of 0.61 (95% confidence interval [CI], 0.51–0.72; *p* < 0.001) [38]. Prespecified analyses further showed that the anti-inflammatory effect of semaglutide was largely independent of both baseline CRP concentration and magnitude of weight loss [40]. Gastrointestinal adverse events were more common with semaglutide, although treatment discontinuation remained relatively low.

In the STEP-HFpEF program echocardiography substudy, semaglutide was associated with favorable changes in selected structural and functional parameters compared with placebo. These data make the clinical findings biologically plausible, but they should be interpreted as hypothesis-generating mechanistic signals rather than proof of disease modification in obesity-related HFpEF [41]. A major strength of STEP-HFpEF was its focus on patient-centered outcomes, particularly symptoms, physical limitations, and functional capacity, which are highly relevant in HFpEF. However, the trial was not powered to assess cardiovascular mortality or heart failure hospitalization as primary clinical endpoints, and the 52-week follow-up limits conclusions regarding long-term remodeling, durability of benefit, and survival.

The SUMMIT trial expanded this therapeutic approach by evaluating the dual glucose-dependent insulinotropic polypeptide (GIP)/GLP-1 receptor agonist tirzepatide in patients with obesity-related HFpEF. The study enrolled adults with HFpEF, defined by LVEF ≥ 50%, obesity, defined as BMI > 30 kg/m^2^, elevated natriuretic peptide concentrations, and symptomatic functional limitation corresponding to NYHA functional class II–IV. Participants were randomized in a phase 3, double-blind, placebo-controlled trial to receive once-weekly tirzepatide, titrated up to 15 mg, or placebo, in addition to background guideline-directed therapy for at least 52 weeks. The dual primary endpoints were a composite of cardiovascular death or worsening heart failure, assessed in a time-to-first-event analysis, and change in KCCQ-CSS from baseline to 52 weeks. Key secondary endpoints included changes in 6MWD, CRP, and body weight [42].

In the ITT population of 731 randomized participants, tirzepatide significantly reduced the composite endpoint of cardiovascular death or worsening heart failure compared with placebo. The reported hazard ratio was 0.62 (95% CI, 0.41–0.95; *p* = 0.026), corresponding to a 38% relative risk reduction in urgent heart failure visits, hospitalization, or cardiovascular death. KCCQ-CSS improved by 19.5 points with tirzepatide compared with 12.7 points with placebo, corresponding to a between-group difference of +6.9 points (95% CI, 3.3–10.6; *p* < 0.001). Body weight decreased by 15.7% with tirzepatide compared with 2.2% with placebo, corresponding to a between-group difference of −13.5%. Tirzepatide also improved functional capacity, increasing 6MWD by approximately 18 m compared with placebo at 52 weeks. Additional secondary analyses demonstrated reductions in inflammatory markers, with an estimated treatment difference of −37.2% (95% CI, −45.7 to −27.3; *p* < 0.001), and in circulatory congestion, reflected by a decrease in estimated blood volume of −0.58 L (95% CI, −0.63 to −0.52; *p* < 0.001) [43,44].

The cardiac magnetic resonance (CMR) substudy of SUMMIT showed that tirzepatide reduced left ventricular (LV) mass and paracardiac adipose tissue compared with placebo, with changes in LV mass paralleling weight loss [45]. SUMMIT’s strengths include longer follow-up and inclusion of harder cardiovascular endpoints that were not primary outcomes in STEP-HFpEF. Importantly, SUMMIT suggests that tirzepatide can reduce worsening HF events and improve symptoms, body weight, congestion-related parameters, and selected structural markers. However, the number of clinical events remained modest, follow-up remains limited for long-term mortality and durable remodeling, and the available data are not sufficient to establish definitive disease modification. As in prior incretin-based studies, gastrointestinal adverse events were the dominant tolerability issue.

Comparisons between STEP-HFpEF and SUMMIT should be interpreted cautiously because the trials differed in inclusion criteria, LVEF thresholds, natriuretic peptide requirements, follow-up duration, endpoint hierarchy, and statistical design. Nevertheless, both trials consistently demonstrated improvements in symptoms, functional capacity, body weight, and obesity-related inflammation, supporting the role of incretin-based therapy as a phenotype-directed strategy in obesity-related HFpEF.

Observational analyses and emerging real-world studies also suggest potential reductions in heart failure hospitalization with incretin-based therapies in patients with obesity-related HFpEF, although residual confounding remains likely. In a large real-world observational study using US claims and electronic health record databases, investigators evaluated the effectiveness of semaglutide and tirzepatide in patients with cardiometabolic HFpEF using a target trial emulation approach modeled after STEP-HFpEF and SUMMIT. Patients initiating semaglutide or tirzepatide were compared with patients receiving sitagliptin after propensity-score-based adjustment to balance baseline clinical characteristics. Over approximately 1 year of follow-up, both semaglutide and tirzepatide were associated with more than 40% lower risk of hospitalization for heart failure or all-cause mortality compared with sitagliptin. Importantly, despite greater weight loss observed with tirzepatide in obesity trials, no significant difference in clinical cardiovascular outcomes was observed between tirzepatide and semaglutide in this HFpEF cohort. Overall, these findings are directionally consistent with STEP-HFpEF and SUMMIT and support the concept that incretin-based therapies may improve clinical outcomes in cardiometabolic HFpEF through mechanisms that include weight reduction, decreased inflammation, reduction in epicardial adiposity, and improved metabolic and diastolic function. However, because this was an observational analysis, residual confounding, treatment-selection bias, and misclassification cannot be fully excluded [46].

Overall, current evidence suggests that GLP-1 RAs and dual incretin therapies represent promising phenotype-directed strategies for obesity-related HFpEF. Semaglutide demonstrated robust improvements in symptoms, body weight, inflammatory markers, and physical function, while tirzepatide additionally showed favorable effects on worsening HF events in SUMMIT. Future studies should clarify durability, background-therapy interactions, combination therapy with SGLT2 inhibitors, comparative effectiveness, and hard long-term outcomes before definitive disease-modifying claims can be made.

To facilitate interpretation across heterogeneous evidence sources, Table 1 summarizes the main cardiovascular outcomes trials, HFpEF-specific randomized trials, ongoing trials, and supportive real-world analyses evaluating GLP-1 receptor agonists and dual incretin therapies in populations with obesity. The table emphasizes key differences in population selection, diabetes status, BMI thresholds, HFpEF definitions, LVEF criteria, follow-up duration, endpoints, and limitations.

## 6. Discussion

The convergence of obesity, systemic inflammation, metabolic dysfunction, and impaired cardiorespiratory reserve has reinforced the concept that obesity-related HFpEF represents a distinct cardiometabolic phenotype rather than simply a higher-body-weight variant of non-phenotyped HFpEF. Excess visceral and epicardial adiposity may promote myocardial stiffness, microvascular dysfunction, endothelial activation, renal sodium retention, pulmonary vascular perturbations, and exercise intolerance, thereby providing a biologically plausible framework for targeted anti-obesity treatment in HFpEF [47,48,49,50].

Within this context, GLP-1 RAs and dual incretin therapies have generated substantial clinical interest because they may favorably influence several pathophysiological mechanisms simultaneously. Their potential value in obesity-related HFpEF is unlikely to be mediated by weight loss alone. Reductions in adipose tissue inflammation, improved insulin sensitivity, lower cardiac filling pressures, attenuation of congestion-related end-organ dysfunction, improved exercise tolerance, and regression of ectopic adipose depots all represent plausible pathways through which incretin-based therapies may improve symptoms and, potentially, clinical cardiovascular outcomes [38,40,49,51,52].

### Mechanistic Interpretation and Scope of Inference

Mechanistic interpretation requires caution because the available mechanistic evidence is not uniform. Weight reduction, lower blood pressure, reduced estimated blood volume, and regression of epicardial or paracardiac adipose tissue are probably weight-loss-dependent or at least strongly linked to adiposity reduction. By contrast, reductions in CRP, NT-proBNP, and markers of congestion are trial-observed biomarkers that may reflect both weight-dependent and weight-independent effects. Effects on myocardial fibrosis, microvascular inflammation, skeletal-muscle quality, renal-adipose crosstalk, and durable reverse remodeling remain biologically plausible hypotheses that require prospective mechanistic studies.

The STEP-HFpEF program reported reductions in systemic inflammation that appeared partly independent of baseline CRP and magnitude of weight loss, while SUMMIT secondary analyses reported reductions in circulatory overload and end-organ damage and the CMR substudy demonstrated lower LV mass and paracardiac adipose tissue. These data support biological plausibility but should not be interpreted as definitive causal proof that incretin therapies modify the natural history of obesity-related HFpEF.

However, interpretation of incretin-based therapy in HFpEF should be situated within the broader CVOT framework. CVOTs have been essential for defining the cardiovascular safety and, in selected cases, cardiovascular efficacy of cardiometabolic therapies. At the same time, they illustrate an important methodological principle: cardiovascular benefits should not be automatically extrapolated from one population to another, or from one pharmacological agent to an entire drug class. This is particularly relevant for GLP-1 RAs and dual incretin therapies, because trials conducted primarily in patients with T2DM cannot be uncritically generalized to patients with obesity, cardiovascular risk, and HFpEF without diabetes. Similarly, evidence generated with one incretin-based agent should not be assumed to apply uniformly to all GLP-1 RAs or to all dual incretin therapies. The sodium-glucose cotransporter 2 (SGLT2) inhibitor CVOT experience provides an instructive precedent: empagliflozin demonstrated superiority for major cardiovascular outcomes in EMPA-REG OUTCOME, whereas dapagliflozin in DECLARE–TIMI 58 fulfilled cardiovascular safety criteria and reduced cardiovascular death or hospitalization for heart failure, but did not reduce MACE compared with placebo [53,54]. These differences emphasize that cardiometabolic trial evidence should be interpreted at the level of agent, dose, population, and endpoint rather than as an undifferentiated class effect [55].

Among currently available agents, semaglutide has the most mature evidence base across obesity treatment, cardiovascular prevention, and HFpEF-specific trials. In STEP-HFpEF, once-weekly semaglutide 2.4 mg significantly improved HF-related symptoms, physical limitations, 6MWD and body weight in patients with obesity-related HFpEF. These findings were clinically important because they addressed outcomes directly relevant to patients with HFpEF, a population often characterized by symptom burden, exertional intolerance and impaired quality of life. The consistency of benefit across prespecified analyses, including NYHA functional class and sex-based analyses, further supports the robustness of the benefit signal [38,56,57].

The STEP-HFpEF DM trial extended this paradigm to patients with obesity-related HFpEF and T2DM, a clinically important subgroup because diabetes is highly prevalent in HFpEF and may amplify inflammation, renal dysfunction, and adverse ventricular–vascular coupling. In that trial, semaglutide again improved KCCQ-based health status, physical limitations, exercise function, and body weight. Accordingly, the STEP-HFpEF program suggests that semaglutide provides reproducible symptomatic and functional benefits across a broad metabolic spectrum of obesity-related HFpEF [51,58,59]. Importantly, the existence of both STEP-HFpEF and STEP-HFpEF DM avoids reliance on extrapolation from diabetes populations alone and supports the need for phenotype- and comorbidity-specific evaluation of incretin-based therapies. Mechanistically, secondary analyses of the STEP-HFpEF program have made the clinical findings more biologically plausible. Semaglutide was associated with reductions in *N*-terminal pro-B-type natriuretic peptide (NT-proBNP) and CRP, supporting potential effects on congestion and systemic inflammation. Echocardiographic analyses suggested favorable cardiac remodeling, while subgroup analyses involving atrial fibrillation, frailty, and diuretic use indicated that benefit was consistent across clinically relevant comorbidity subpopulations. Taken together, these observations support the concept that semaglutide may also act directly on core components of obesity-related HFpEF pathophysiology rather than producing body-weight reduction alone [40,41,52,60,61].

The broader cardiovascular relevance of semaglutide was demonstrated by SELECT, in which semaglutide reduced MACE in patients with overweight or obesity and established cardiovascular disease, but without diabetes. Although SELECT was not designed specifically as an HFpEF trial, it was highly informative because it demonstrated that pharmacological treatment of obesity can translate into fewer ischemic cardiovascular events in a non-diabetic population. Moreover, a prespecified analysis showed that semaglutide reduced the composite of cardiovascular death or heart failure events in participants with prevalent heart failure, strengthening the argument that the cardiovascular value of semaglutide in obesity extends beyond symptom control alone [33,62].

Nevertheless, semaglutide’s evidence profile in obesity-related HFpEF requires appropriate nuance. The STEP-HFpEF trials were centered principally on symptoms, functional capacity, body weight, and biomarkers rather than adjudicated long-term heart failure outcomes, whereas SELECT enrolled a broad population with established cardiovascular disease rather than a dedicated HFpEF cohort. Thus, semaglutide can currently be viewed as the agent with the strongest integrated evidence for improving clinical status in obesity-related HFpEF and for reducing atherosclerotic cardiovascular events in patients with overweight or obesity. However, it has not yet been tested in a dedicated HFpEF outcomes trial powered primarily for long-term heart failure events or cardiovascular mortality [33,38,51,62].

Dual incretin therapy may further expand this therapeutic field. Tirzepatide, through combined glucose-dependent insulinotropic polypeptide and GLP-1 receptor agonism, appears particularly attractive for cardiometabolic HFpEF because of its potent effects on body weight and central adiposity. In SUMMIT, tirzepatide reduced the composite of cardiovascular death or worsening heart failure and improved health status in patients with HFpEF and obesity. This trial marked an important advance because it moved beyond symptom improvement alone and provided dedicated outcome-trial evidence in the obesity-related HFpEF phenotype [42]. Substudies and follow-up analyses from SUMMIT added mechanistic depth to the primary findings. Tirzepatide reduced indices of circulatory congestion and end-organ damage, while cardiac magnetic resonance imaging demonstrated regression of left ventricular mass together with reductions in paracardiac adipose tissue. These effects are highly relevant in obesity-related HFpEF, where ventricular interdependence, pericardial restraint, and adipose-mediated inflammatory signaling may contribute meaningfully to symptom worsening and disease progression. Such findings support the hypothesis that dual incretin therapy may influence selected structural features of cardiometabolic HFpEF, but they should be viewed as supportive mechanistic signals rather than definitive evidence of disease modification [43,45].

A central unresolved question is whether dual incretin therapy is intrinsically superior to GLP-1 receptor agonism in obesity-related HFpEF, or whether the apparent incremental benefit of tirzepatide mainly reflects greater weight loss and adipose tissue reduction. Cross-trial comparisons are inherently limited by differences in eligibility criteria, background therapies, endpoint definitions, event ascertainment, follow-up duration, baseline comorbidity burden, and trial conduct. Therefore, the available evidence should not be interpreted as establishing a definitive class hierarchy across incretin-based therapies. Rather, the current evidence base supports distinct clinical observations for individual agents, while precluding reliable conclusions regarding their relative efficacy in HFpEF. Semaglutide has the most established cross-platform evidence spanning obesity treatment, HFpEF symptom improvement, and atherosclerotic cardiovascular risk reduction, whereas tirzepatide has shown the most direct signal for reducing worsening heart failure events in a dedicated obesity-related HFpEF outcomes trial [32,33,38,42,51,55,62]. However, in the absence of direct comparative evidence, these findings should not be interpreted as indicating superiority of either agent for HFpEF management.

From a clinical standpoint, incretin-based therapies should be integrated with, rather than substituted for, established HFpEF care. SGLT2 inhibitors have appropriately become foundational therapy in HFpEF and HFmrEF, while diuretics remain essential for congestion relief; blood-pressure control, rhythm and rate management for atrial fibrillation, ischemia management, sleep-apnea treatment, renal protection, diabetes care, exercise rehabilitation, nutritional support, and structured weight management remain core components of care [27,63,64]. Incretin-based therapies may therefore evolve as phenotype-directed add-on treatments for patients with obesity-dominant cardiometabolic HFpEF, particularly when excess adiposity, prediabetes or diabetes, systemic inflammation, and congestion coexist. Combination therapy with SGLT2 inhibitors is biologically attractive because these drug classes target partly complementary pathways, but STEP-HFpEF and SUMMIT were not designed to establish optimal sequencing, additive efficacy, or safety of routine combination therapy. Dedicated trials and prespecified subgroup analyses according to background SGLT2 inhibitor use are needed.

Available subgroup data suggest generally consistent symptomatic and functional benefits across diabetes status when STEP-HFpEF and STEP-HFpEF DM are considered together, and across sex, NYHA functional class, frailty, atrial fibrillation, and diuretic-use subgroups within the STEP-HFpEF program. However, these analyses were not powered to establish treatment heterogeneity according to baseline BMI, natriuretic peptide concentration, renal function, atrial fibrillation, congestion burden, or background HFpEF therapy. Differential response by these variables remains an important future research priority.

Several caveats remain. Obesity-related HFpEF is biologically heterogeneous, and BMI alone is an imperfect surrogate for the pathophysiological burden of visceral and epicardial adiposity. Longer-term data are still needed regarding durability of benefit, treatment persistence, lean-mass preservation, tolerability, cost-effectiveness, and integration with established heart failure-directed therapies. In this context, future studies should move beyond body weight and BMI as the dominant anthropometric endpoints. Although weight reduction may improve symptoms, congestion, inflammation, and exercise tolerance, total body weight does not distinguish between loss of adipose tissue, lean mass, or skeletal muscle quality. This distinction is particularly important in HFpEF, where sarcopenic obesity, frailty, impaired peripheral oxygen utilization, and reduced skeletal muscle function may contribute substantially to exercise intolerance and long-term outcomes. Therefore, future trials of GLP-1 RAs and dual incretin therapies in obesity-related HFpEF should systematically assess body composition and physical performance, including dual-energy X-ray absorptiometry for fat mass and lean mass, CT- or MRI-based quantification of visceral adiposity, epicardial or paracardiac adipose tissue, and skeletal muscle mass or quality, as well as functional measures such as handgrip strength, gait speed, frailty indices, and cardiopulmonary exercise testing. Incorporating these endpoints would help clarify whether clinical benefits are mediated predominantly by adipose tissue reduction, improved congestion and inflammation, preservation or loss of lean mass, or changes in skeletal muscle function.

Therefore, future trials should prioritize clinically meaningful multidimensional endpoints rather than total body weight reduction alone, integrating functional capacity, symptoms, health-related quality of life, and cardiovascular outcomes with quantitative assessment of visceral/epicardial adiposity, skeletal muscle mass and quality, frailty, and lean mass preservation.

Furthermore, whether improvements in symptoms and worsening heart failure events will translate into clear reductions in cardiovascular mortality across broader real-world HFpEF populations remains uncertain. These considerations should restrain overgeneralization while not diminishing the genuine therapeutic progress represented by current trials [42,48,49,63]. Future research should move beyond proof-of-concept efficacy and prioritize long-term cardiovascular outcomes of GLP-1 RAs and dual incretin therapies in patients with obesity, cardiovascular risk, and rigorously defined HFpEF. In parallel, trials should test whether treatment response differs across obesity phenotypes, including sarcopenic obesity and visceral or epicardial adiposity-dominant forms, because BMI alone incompletely captures the cardiometabolic substrate most relevant to obesity-related HFpEF. Phenotype-guided approaches may enable more precise patient selection and more rational deployment of incretin-based therapy [65]. Importantly, future trials should also distinguish HFpEF, usually defined by LVEF ≥ 50%, from HFmrEF, usually defined by LVEF 41–49%, because these populations differ in etiology, pathophysiology, prognosis, and treatment responsiveness [39].

Head-to-head randomized trials comparing GLP-1 RAs with dual incretin therapies are also needed to define relative efficacy, cardiovascular protection, tolerability, metabolic effects, and cost-effectiveness. At present, comparisons between semaglutide and tirzepatide remain largely indirect and are therefore methodologically fragile, because available trials differ substantially in baseline population, comorbidity profile, inclusion and exclusion criteria, endpoint definitions, follow-up duration, background therapy, and statistical hierarchy. Such heterogeneity limits external validity and may introduce systematic bias when treatments are compared across rather than within trials [32,55]. Carefully designed head-to-head trials, ideally complemented by pragmatic registry-based randomized studies and high-quality observational cohorts, would provide more clinically actionable evidence.

Across these evidence strata, clinical inference should be drawn at the level of individual agent, dose, population, background therapy, and endpoint. Diabetes CVOTs support cardiovascular safety and selected benefits in T2DM; SELECT supports semaglutide for secondary cardiovascular prevention in obesity without diabetes; STEP-HFpEF and SUMMIT support symptom, function, body-weight, biomarker, and selected HF-event benefits in obesity-related HFpEF; mechanistic substudies provide biological plausibility; and observational studies remain hypothesis-generating despite sophisticated causal emulation methods. This hierarchy should guide interpretation and prevent overextension of findings across populations or drug classes.

Future CVOT methodology should also evolve. The conventional time-to-first-event approach remains widely used and familiar to regulators, but it may be insufficient for complex cardiometabolic syndromes such as obesity-related HFpEF. In a standard composite endpoint, a first hospitalization for worsening heart failure and cardiovascular death may be treated as competing components of the same endpoint, although they differ substantially in clinical severity and patient relevance. Similarly, time-to-first-event analyses may fail to capture recurrent events, functional improvement, symptom burden, treatment-related burden, and quality-of-life gains. Hierarchical composite outcomes, win-ratio methods, and win-odds approaches may therefore provide a more clinically coherent framework by ranking outcomes according to their importance while still incorporating multidimensional treatment effects [55,66]. These methods may be particularly useful in future incretin-based CVOTs, where benefits may span cardiovascular events, heart failure progression, weight loss, functional capacity, symptoms, and health-related quality of life. Patient-centered outcomes should remain central. Functional capacity, symptom burden, exercise tolerance, treatment burden, and health-related quality of life complement conventional MACE and heart failure hospitalization endpoints and are especially relevant in HFpEF, where disability and exertional intolerance are often dominant clinical problems [67]. Looking further ahead, digital twin technologies may offer opportunities to model individualized treatment responses, enrich trial design, and potentially serve as virtual comparators or exploratory trial arms, provided they become sufficiently accurate, clinically validated, and externally reproducible [68].

Mechanistic studies should accompany these clinical trials to clarify how GLP-1 RAs and dual incretin therapies affect myocardial remodeling, systemic and cardiac inflammation, congestion biology, skeletal muscle quality, renal–adipose interactions, and metabolic regulation. Together, these priorities define a roadmap for obesity-related HFpEF that should encompass phenotype-specific investigation, agent-specific rather than class-level interpretation, rigorous head-to-head comparisons, pragmatic trial methodology, mechanistic imaging and biomarker studies, patient-centered endpoints, and carefully validated digital applications.

## 7. Conclusions

Obesity-related HFpEF is increasingly recognized as a distinct cardiometabolic syndrome in which adiposity is not merely a comorbidity, but a potentially modifiable disease substrate. Current evidence indicates that incretin-based therapies can meaningfully improve symptoms, functional capacity, body weight, congestion-related biomarkers, systemic inflammation, and selected cardiovascular outcomes in carefully selected patients with obesity and HFpEF. Semaglutide currently provides the strongest integrated evidence across obesity treatment, HFpEF health-status improvement, and cardiovascular risk reduction in individuals without diabetes, whereas tirzepatide has extended the field by demonstrating a clinically important reduction in cardiovascular death or worsening heart failure in SUMMIT, a dedicated obesity-related HFpEF trial. However, given the modest number of clinical events and the current duration of follow-up, these findings support the possibility of disease-modifying effects, but do not establish definitive long-term disease modification or cardiovascular mortality reduction.

However, these data should be interpreted with methodological caution. Findings from T2DM CVOTs should not be extrapolated uncritically to obesity-related HFpEF without diabetes, and conclusions should be drawn at the level of individual agents, populations, doses, background therapies, and endpoints rather than assumed to reflect uniform class effects. Future trials should prioritize long-term cardiovascular outcomes, direct head-to-head comparisons, combination strategies with SGLT2 inhibitors, phenotype-specific enrollment, clear separation of HFpEF from HFmrEF, and more clinically meaningful hierarchical endpoint frameworks. Such an approach will be essential to determine whether GLP-1 RAs and dual incretin therapies can become durable outcome-modifying components of multimodal obesity-related HFpEF care rather than only symptom- and weight-management interventions.

## Figures and Tables

**Table 1:** Main studies of GLP-1 receptor agonists and dual incretin therapies across cardiovascular outcomes and obesity-related HFpEF evidence.

Study	Evidence Category	Drug	Comparator	Population	Diabetes Status	BMI Criteria	HFpEF Definition/LVEF	Sample Size	Follow-Up	Primary Endpoint	Main Findings	Key Limitations
SELECT	CVOT; obesity without diabetes	Semaglutide 2.4 mg weekly	Placebo	Adults ≥ 45 years with overweight/obesity and established CVD	Excluded diabetes	BMI ≥ 27 kg/m^2^	Not HFpEF-specific	17,604	Mean follow-up 39.8 ± 9.4 months	CV death, nonfatal MI, or nonfatal stroke	Reduced 3-point MACE; HR 0.80; 6.5% vs. 8.0%	Secondary prevention population; not designed as HFpEF trial; mechanisms of CV benefit not fully separable from weight loss
SURMOUNT-MMO	Ongoing CVOT; obesity without diabetes	Tirzepatide up to 15 mg weekly	Placebo	Adults ≥ 40 years with obesity/overweight and established CVD or multiple CV risk factors	Excluded diabetes	BMI ≥ 27 kg/m^2^	Not HFpEF-specific	~15,000 planned	Event-driven; up to ~5 years	Time to first nonfatal MI, nonfatal stroke, coronary revascularization, HF event, or all-cause death	Ongoing; no efficacy results yet	Results pending; endpoint heterogeneity; not a dedicated HFpEF population
STEP-HFpEF	HFpEF-specific RCT	Semaglutide 2.4 mg weekly	Placebo	Symptomatic obesity-related HFpEF	Without diabetes/non-DM HFpEF cohort	BMI ≥ 30 kg/m^2^	HFpEF with LVEF ≥ 45%; NYHA II–IV	529	52 weeks	Dual primary endpoints: change in KCCQ-CSS and percent body weight	Improved KCCQ-CSS, 6MWD, body weight, and CRP	Not powered for CV death or HF hospitalization; 52-week duration; symptom/function-centered endpoints
SUMMIT	HFpEF-specific outcomes RCT	Tirzepatide up to 15 mg weekly	Placebo	Symptomatic HFpEF with obesity	Included patients with and without diabetes, depending on trial criteria	BMI ≥ 30 kg/m^2^	HFpEF with LVEF ≥ 50%	731	Median 104 weeks	Composite of CV death or worsening HF; change in KCCQ-CSS	Reduced CV death/worsening HF; HR 0.62; improved KCCQ-CSS and body weight	Modest number of clinical events; cross-trial comparison with semaglutide not valid; mechanisms remain difficult to separate from weight loss
STEER	Real-world observational evidence	Semaglutide	Tirzepatide	US insured adults ≥ 45 years with overweight/obesity and established ASCVD	Excluded diabetes	Overweight/obesity; exact BMI criteria should follow original report	Not HFpEF-specific	Semaglutide 14,483; tirzepatide 10,642 before matching	Short real-world follow-up	MACE outcomes, including MACE-3 and MACE-5	Semaglutide associated with lower MACE-3 and MACE-5 vs. tirzepatide in observational analyses	Non-randomized; residual confounding; treatment-selection bias; short follow-up; claims-based outcome ascertainment

Abbreviations: ASCVD, atherosclerotic cardiovascular disease; BMI, body mass index; CRP, C-reactive protein; CV, cardiovascular; CVD, cardiovascular disease; CVOT, cardiovascular outcomes trial; HF, heart failure; HFpEF, heart failure with preserved ejection fraction; HR, hazard ratio; KCCQ-CSS, Kansas City Cardiomyopathy Questionnaire Clinical Summary Score; LVEF, left ventricular ejection fraction; MACE, major adverse cardiovascular events; MI, myocardial infarction; RCT, randomized controlled trial; 6MWD, 6-minute walk distance.

## Data Availability

No new data were created or analyzed in this study. Data sharing is not applicable to this article.
